# Loop dynamics of thymidine diphosphate-rhamnose 3′-*O*-methyltransferase (CalS11), an enzyme in calicheamicin biosynthesis

**DOI:** 10.1063/1.4941368

**Published:** 2016-02-18

**Authors:** Lu Han, Shanteri Singh, Jon S. Thorson, George N. Phillips

**Affiliations:** 1 Biosciences at Rice, Rice University, Houston, Texas 77005, USA; 2 Center for Pharmaceutical Research and Innovation, Pharmaceutical Sciences, University of Kentucky College of Pharmacy, Lexington, Kentucky 40536-0596, USA; 3 Chemistry Department, Rice University, Houston, Texas 77005, USA

## Abstract

Structure analysis and ensemble refinement of the apo-structure of thymidine diphosphate (TDP)-rhamnose 3′-*O*-methyltransferase reveal a gate for substrate entry and product release. TDP-rhamnose 3′-*O*-methyltransferase (CalS11) catalyses a 3′-*O*-methylation of TDP-rhamnose, an intermediate in the biosynthesis of enediyne antitumor antibiotic calicheamicin. CalS11 operates at the sugar nucleotide stage prior to glycosylation step. Here, we present the crystal structure of the apo form of CalS11 at 1.89 Å resolution. We propose that the L2 loop functions as a gate facilitating and/or providing specificity for substrate entry or promoting product release. Ensemble refinement analysis slightly improves the crystallographic refinement statistics and furthermore provides a compelling way to visualize the dynamic model of loop L2, supporting the understanding of its proposed role in catalysis.

## I. INTRODUCTION

**FIG. 1.**
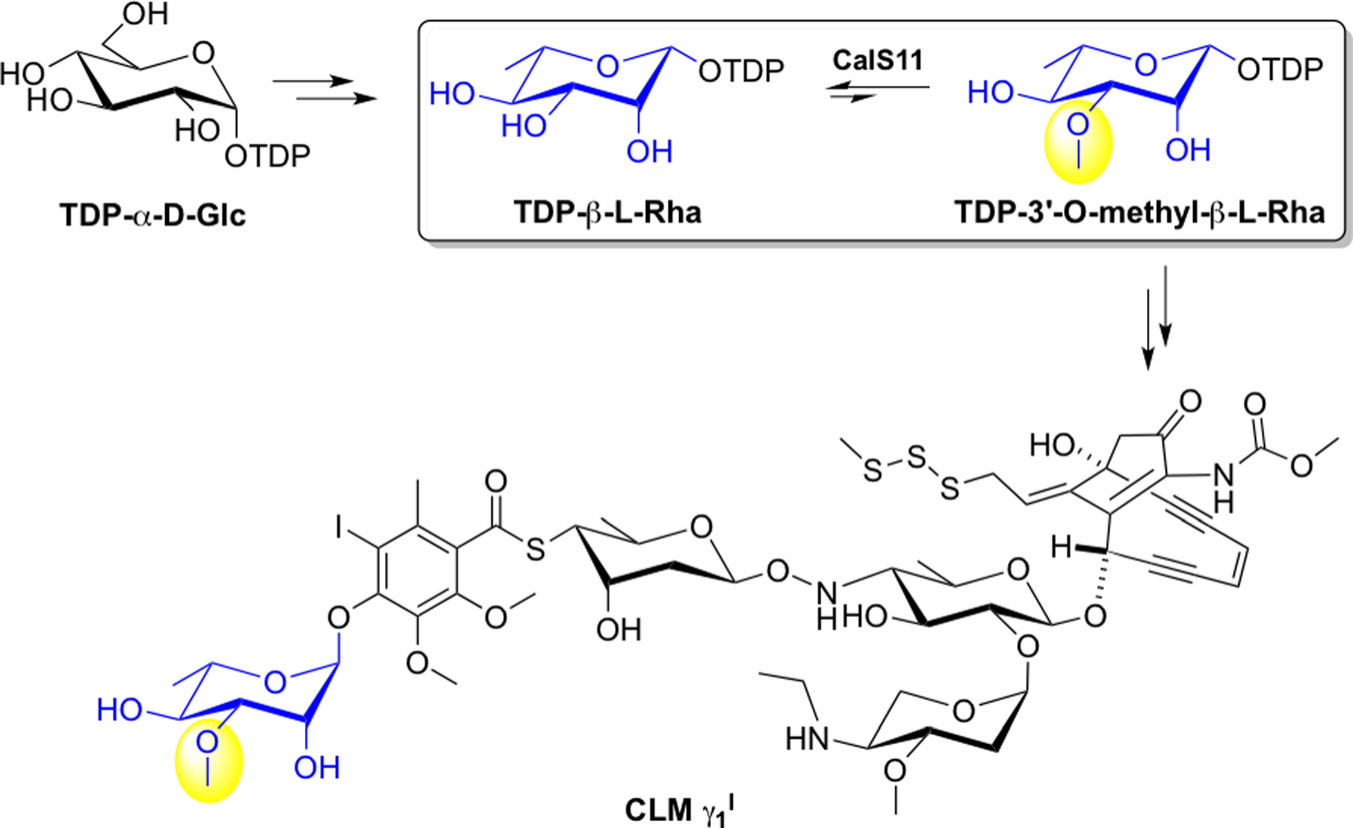
The biosynthetic pathway of TDP-methoxy-rhamnose in *M. echinospora* en route to calicheamicin _ϒ1_^I^ production.

Natural products remain invaluable sources for drug leads and bioactive probes.^1,2^ Discovering new mechanisms for the biosynthesis of important natural products and exploiting knowledge of natural product biosynthesis enzymes could help produce new diversified biosynthetic or semisynthetic natural products for various purposes.^3–6^ As part of the NIH Protein Structure Initiative, a high-throughput structural genomics approach has been employed to clone, express, purify, and solve structures of novel enzymes for natural product biosynthesis.^7–18^ One targeted pathway for this initiative has been that leading to the biosynthesis of calicheamicin (CLM), a 10-membered enediyne antitumor antibiotic produced by *Micromonospora echinospora*.^19,20^ Upon bioreduction, CLM undergoes a Bergman-type cyclization reaction, the benzene diradical species of which lead to DNA backbone hydrogen abstraction and subsequent irreparable oxidative DNA strand scission.^21,22^ CalS11, a protein encoded by the calicheamicin biosynthetic gene locus,^23,24^ catalyzes a late-stage glycosyl tailoring event (thymidine diphosphate (TDP)-L-rhamnose 3′-*O*-methylation) prior to glycosyltransferase (CalG1)-catalyzed transfer to complete aryltetrasaccharide assembly (Figure 1).^10,25^ Like all prototypical class I methyltransferases,^26^ CalS11 uses *S*-adenosylmethionine (AdoMet, SAM) as the methyl donor. However, CalS11 is distinguished from other sugar *O*-methyltransferases by virtue of its activity at the sugar nucleotide prior to glycosyltransfer.^10,27^

Structural flexibility and dynamics are generally key for protein function.^28^ Functionally important motions not only involve ordered secondary structures but more commonly involve disordered loop structures. As shown by the study on CalS11 and many other proteins, such as xylanase protein from *Thermobacillus xylanilyticus*,^29^ loop dynamics are important for substrate binding and product release. Loop dynamics are also important for allosteric activation of enzymes, such as kinases and tyrosine phosphatases.^30^ Loop dynamics are also widely found in eukaryotic regulatory proteins involved in processes such as signal transduction and transcription, allowing for an induced fit molecular recognition process.

However, prevailing static models are simply an average of an ensemble of states and cannot adequately describe the dynamics of protein molecules. Ensemble refinements (ERs) have been developed that use the X-ray diffraction data to generate an ensemble of models to represent a non-Gaussian distribution of positions and imply the corresponding motions of the protein molecules. This concept was first proposed two decades ago by Brunger and Kuriyan^31^ and was extended and tested by several other groups of scientists.^32,33^ Burnley *et al.* developed an implementation (phenix.ensemble_refinement) as part of the Phenix software package^33^ which lowers the barrier for others to use this approach. Starting from a well-refined single model, local molecular vibrations and rotations are sampled by molecular dynamics (MD) simulation restrained with terms incorporating the X-ray data, while global disorder is partitioned into an overall translation-libration-screw (TLS) model.^33^ Large numbers of structures make up the ensemble, typically thousands, but in the end, a small number of structures that reproduce the best R_free_ within some tolerance, typically 0.1%, are kept as the final representative set of structures defining the ensemble. We have applied ER techniques to model both the structure and dynamics based on X-ray diffraction data sets for both *S*-adenosylhomocysteine (SAH) bound and apo-structures. The analysis shows the L2 loop is indeed highly flexible in ways that are consistent with enzymatic turnover, whereas the highly conserved loops L1 and L3 have dramatically more stable structures.

We previously solved and reported two structures of the SAH bound form of CalS11 [Protein Databank (PDB) entry 3TOS, 4GF5]^10^ and have now solved the corresponding apo structure of CalS11 at 1.89 Å resolution (PDB entry 4PWR). Compared to its substrate bound structure,^10^ where loop L2 is closed over the SAH, the electron density for loop L2 in the apo-form is inadequate for establishing a static model, reflecting the dynamic, or at least disordered nature of substructure L2. The observed structural difference in the states of CalS11 with and without SAH bound prompted further evaluation of the structural dynamics of CalS11 in its catalytic function.

## II. MATERIALS AND METHODS

### A. Crystallization and data collection and refinement

Protein cloning, expression, and purification methods were performed as previously described.^10^ Apo CalS11 crystals were grown with hanging drop vapor diffusion method by mixing 1 *μ*l of protein solution (16 mg/ml CalS11 in 25 mM tris, pH 8.0) and 1 *μ*l reservoir solution (25% polyethylene glycol 3350, 0.2 M Li_2_SO_4_, 0.1 M Bis-Tris pH 6.5). The crystal was flash frozen in liquid nitrogen for data collection without additional cryoprotectants. Diffraction data were collected at APS 21-ID-D beamline and were processed with XDS.^34^ The apo structure was solved by molecular replacement using phaser-MR from Phenix suite^35^ with molecule A from CalS11 complex structure (PDB 4GF5) as the search model. The model was improved by alternating cycles of manual model building using Coot^36^ and refinement using Phenix. Visual analysis of the final difference maps and interpretation of the structure was performed with a collaborative stereoscopic system based on a commodity 3D television.^37^ The final model was validated using MolProbity^38^ and deposited in the Protein Data Bank with accession code 4PWR.

### B. Ensemble refinement of CalS11

We performed refinement using the scripts within Phenix.ensemble_refinement for both the substrate bound (PDB 3TOS, 4GF5)^10^ and unbound structures of CalS11 (PDB 4PWR). For the substrate bound structure, the downloaded PDB files were refined using TLS refinement before being used as input file for ensemble refinement. For the apo CalS11 structure, after regular phenix.refine step, the missing region of L2 was arbitrarily built in manually with correct sequence and stereochemistry and was subsequently used as input for the ensemble refinement. We limited the number of models to be used to prevent over fitting of the data.^33^ Harmonic restraints were applied for all amino acids with visible electron density at a level of 1σ in the 2mFo-Dfc electron density map using parameters weight = 0.0001 and slack = 1.0, as suggested in the documentation.

## III. RESULTS AND DISCUSSION

### A. Overall structure of apo CalS11

**FIG. 2.**
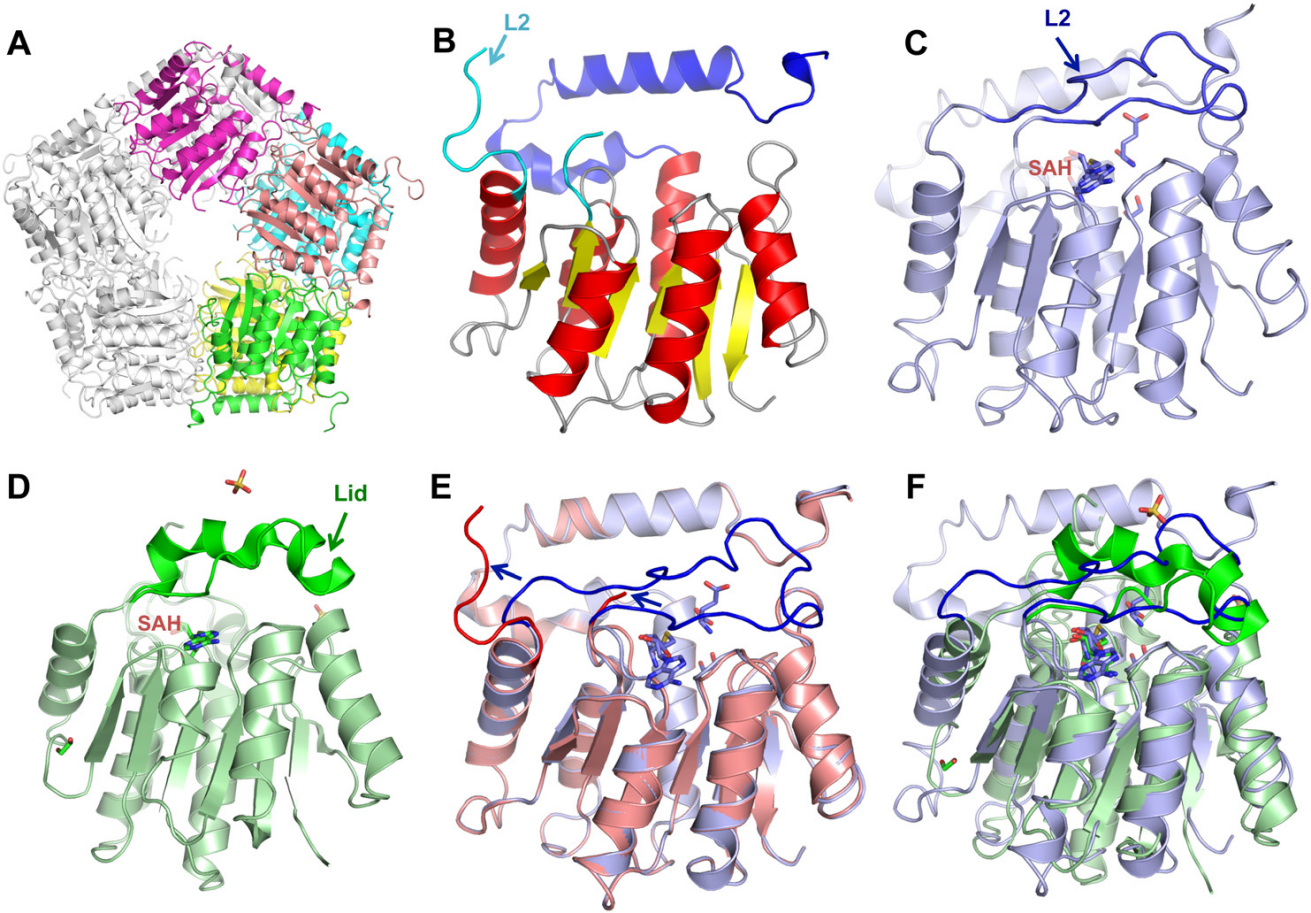
(a) The apo form of CalS11 forms a decamer in solution. Each asymmetric unit contains half of a decamer (shown with colors). (b) Apo CalS11 monomer with secondary structural element, L2, labelled. (c) The SAH/glutamate complex CalS11 monomer structure (PDB 3TOS^10^). (d) The SAH complex NovP monomer structure (PDB 2WK1^39^). (e) Superimposition of apo (red) and complexed (blue) CalS11 structures. (f) Superimposition of NovP structure (green) and CalS11 complexed structure (blue).

The structure of the apo CalS11 crystal was determined at a nominal resolution of 1.89 Å. This structure belongs to space group C2, different from previously reported space groups of CalS11, P1. The final structure was refined to R_cryst_ and R_free_ of 13.3% and 16.8% (PDB entry 4PWR) (Table I). Each CalS11 monomer folds in the same way as the substrate bound form (Figure 2(b)), into a single globular domain comprising a Rossmann fold characteristic of all SAM-dependent methyltransferases. Apo CalS11 also forms a decamer of five interconnected dimers. Each asymmetric unit contains half of the functional decamer (Figure 2(a)). In the search results for similar structures, NovP, the novobiocin L-noivose-4′-O-methyltransferase is the closest structure available. Different from CalS11's decameric structure, NovP exists as dimer in solution. The C-alpha coordinates root-mean-square-deviation (r.m.s.d.) of NovP aligned with CalS11 is 3.2 Å for the bound structure and 1.9 Å for the apo structure. The sequence identity of NovP and CalS11 is 20%. The Rossmann fold part of the structures aligns well, while the L2 regions of these two structures are fairly distinct.

**TABLE I.** Statistics for data collection and refinement of the crystal structure of CalS11. Values in parenthesis are for the highest resolution shell.

PDB ID	4PWR
Spacegroup	C 1 2 1
Wavelength (Å)	0.9787
Unit cell parameters	148.26 125.14 107.15 90.00 125.12 90.00
Estimated standard deviation of cell parameters	0.14 0.09 0.07 0.00 0.03 0.00
Resolution range of data collection (Å)	47.85 − 1.793 (1.857 − 1.793)
No. of reflections (measured/unique)	1 098 192/147 725 (91 275/13 326)
Completeness % (Å)Multiplicity	99 (90)7.4 (6.8)
Mean I/sigma(I)	10.25(1.39)
Wilson B-factor	25.40
R-merge^a^	0.1316 (1.234)
R-meas^b^	0.1415 (1.332)
CC1/2	0.997 (0.611)
CC*	0.999 (0.871)
R-cryst^c^	0.146 (0.298)
R-free^d^	0.179 (0.317)
Number of non-hydrogen atoms	11 176
Macromolecules	9403
Ligands	25
Protein residues	1162
RMS(bonds)	0.01
RMS(angles)	1.0
Ramachandran^e^ favored (%)	97.0
Ramachandran outliers (%)	0.09
Rotamer outliers (%)	0.69
Clashscore	3.15
Average B-factor	32.8
Macromolecules	30.7
Ligands	64.1
Solvent	43.24

^a^ *R-merge* = ∑_*hkl*_ ∑_*j*_|*I*_hkl,j_ − ⟨*I*_hkl_⟩|/∑_*hkl*_ ∑_*j*_ *I*_hkl,j_, where ⟨*I*_hkl_⟩ is the average of symmetry related observation of a unique reflection.

^b^ *R-meas* = ∑_*hkl*_ $\sqrt{n/(n-1)}$∑_*j*_|*I*_hkl,j_ − ⟨*I*_hkl_⟩|/∑_*hkl*_ ∑_*j*_ *I*_hkl,j_, which is redundancy independent version of R-merge.

^c^ *R-cryst* = ∑_*hkl*_ ||*F*_obs_| − |*F*_calc_||/∑_*hkl*_ |*F*_obs_|, where *F*_obs_ and *F*_calc_ are the observed and calculated structure-factor amplitudes.

^d^ *R-free* was calculated as *R-work* using randomly selected 5% of the unique reflections that were omitted from the structure refinement.

^e^ Ramachandran statistics indicate the percentage of residues in the most favored, additionally allowed, and outlier regions of the Ramachandran diagram as defined by MOLPROBITY.

Out of the 257 residues in the apo form of CalS11, 6 N-terminal residues and the residues 111–131 are missing in the electron density. It is unlikely that this stretch of protein chain has been cleaved, since these residues are visible in the substrate-bound structures. Thus, the most likely possibility is that the L2 region of CalS11, residues 111–131, undergoes large conformational changes when no substrate is present. The L2 region is adjacent to the substrate-binding cavity. It is likely that the dynamics of L2 facilitates substrate entry and product release. Large conformational changes of L2 are accommodated despite the decameric structure of the enzyme as this loop is on the surface of the decamer.

### B. Active site of apo CalS11

**FIG. 3.**
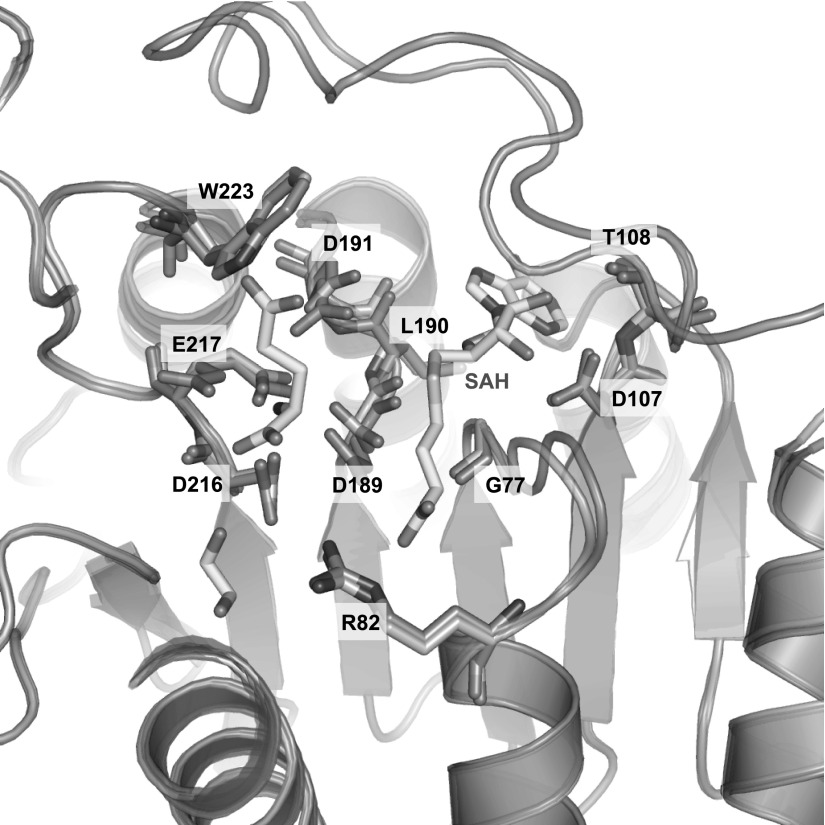
(a) The residues involved in SAH and glutamate binding (substrates in yellow). The apo CalS11 structure is colored red (PDB 4PWR), complex structure is colored blue (PDB 3TOS).

The CalS11 substrate bound structure shows that the SAM/SAH binding site of CalS11 is located in the C-terminal end of the cleft formed by the central β strands. Interactions between CalS11 and the bound SAH as well as the substrate surrogate, glutamate, are mainly provided by residues in three loops (L1, L2, L3), which are conserved in the methyltransferase family. The conformation of these residues of L1 (between β1 and α4) and L3 (between β3 and helix α6) in the apo structure is mostly the same as they are in the complex structure, including the putative CalS11 catalytic base Asp191 (Figure 3). Though many residues of L2 (between β2 and α5) are also conserved, residues 111–128 (or residues 111–132 in some chains) are not visible in the apo structure. In 2 copies out of 5 in asymmetric unit of the apo structure, residues 127–133 of L2 are shifted away from the SAM/SAH site compared to that in the complex structure (Figure 2(e)). In the other 3 copies in the ASU, only residues 132–133 are visible and they show same conformation as the complex structures do. This variability amongst the members of the decamer further support the hypothesis that L2 is flexible and it can either stay close to or move away from SAM/SAH site.

In the CalS11 structurally related protein, NovP, the substructure corresponding to L2 is a half helix-half loop structure. It is also proposed to be flexible, forming a lid over the co-substrate SAM serving as a gate (Figures 2(c) and 2(d)).^39^ This suggests that L2 probably serves a similar function in both NovP and CalS11. In the complex structure, L2 forms a hydrophobic lid near the ribose and adenine ring of SAH and stabilizes SAH by van der Waals forces and sterically hinders the release of SAH. Thus, the dynamics of the loop as observed here may promote SAH release to complete the catalytic cycle.

### C. Ensemble refinements

The ensemble refinement as applied here was performed using an X-ray data-restrained time-averaged molecular dynamics simulation to generate an ensemble of models to represent the special distribution and implies motion within protein molecules. It is an excellent tool to study structural dynamics. Application of ensemble refinement method to all three CalS11 structures improves the agreement between model and x-ray diffraction data, represented by decreases in R_free_ compared to regular refinement (Table II). Application of the ensemble refinement to apo CalS11 structure does reduce the r.m.s.d. of the mF_o_-DF_m_ difference map by 10%, although the ways the absolute scale is calculated are slightly different in the two methods and may or may not account for this difference. Taken together, these results provide some evidence that the ensemble refinement improves model quality and supports the idea that an ensemble of models somewhat better represents the structural dynamics of CalS11 than a single static model. It certainly conveys a better visual description of the conformational variability than does simply removing parts of the model.

**TABLE II.** Statistics of ensemble refinement.

PDB ID	Resolution (Å)	Number of models	phenix.refine		ensemble.refinement (ER)		ER—phenix.refine	
			R_cryst_	R_free_	R_cryst_	R_free_	ΔR_cryst_	ΔR_free_
4PWR	1.80	25	0.146	0.179	0.137	0.171	−0.009	−0.008
3TOS	1.55	20	0.166	0.195	0.138	0.172	−0.028	−0.023
4GF5	2.20	20	0.220	0.219	0.143	0.199	−0.077	−0.020

**FIG. 4.**
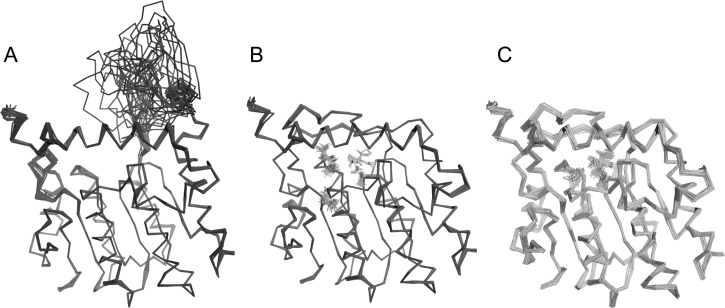
Comparison of ensemble models of apo CalS11 (PDBID 4PWR) and tertiary complexes CalS11 (PDBID 3TOS and PDBID 4GF5). Protein regions with stable conformations show small displacements in these ensemble models, while disordered regions show large displacements. (a) The L2 region of apo CalS11 shows large displacements, while the most of the structure shows small displacements. (b) and (c) Ensemble models of complexed CalS11 (PDBID 3TOS) and CalS11 (PDBID 4GF5) show small displacements, including the L2 region. These results show that the substrate binding site is more solvent accessible when no substrate is bound. All structures are shown in ribbon form, with substrates shown in lines using PYMOL.^41^

**FIG. 5.**
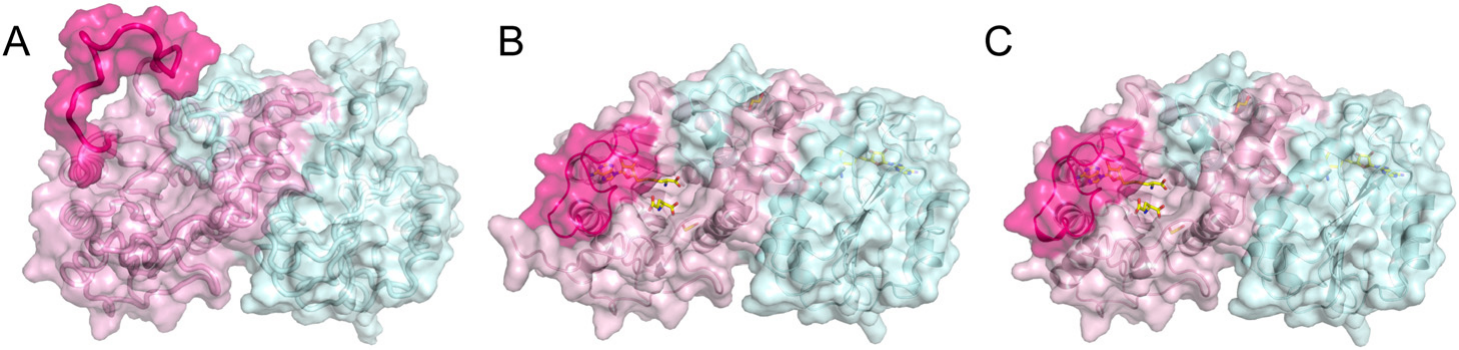
Active site accessibility comparison of three structures of CalS11: 4PWR (a), 3TOS (b), and 4GF5 (c). In all three panels, two subunits were coloured pink and cyan, separately. L2 of the pink subunit is highlighted in hot pink. When substrate is bound (PDBID 3TOS, 4GF5), L2 covers the majority of the active site leaving little opening. When substrate is not bound (a), the cavity can be completely exposed to solvent in many conformations.

The ensemble refinement results for product bound structures are consistent with the standard structure determinations, with L2 staying in the closed state (Figures 4(b) and 4(c)). However, in the ensemble refinement results for the apo structure, L2 is spatially distributed between closed and open conformations (Figure 4(a)). The ensemble of L2 conformations did not form a contact with neighboring unit cell. This indicates that the space displacement range of L2 is not an artifact due to crystal packing. The active site is more exposed to solvent than in the SAH bound structure as a result of L2 movement (Figure 5). Residues involving hydrophobic interactions with SAH (residues 111–113 and residues 128–133) show both main chain and side chain conformation changes.

This mobility of L2 that we see in CalS11 appears to minimize the activation energy for substrate binding, as the loop rearrangements open the conformation for substrate access. The loops interactions with the substrate may also provide specificity for the substrate through enthalpic interactions in intermediate states. If the energy landscape of a loop is very broad, loop variability can also contribute an entropic component to the overall free energy of binding that trades specificity or tightness of binding of products with conformational entropy. This entropic component of the free energy is then recovered as the product leaves the active site.

## IV. CONCLUSIONS

Dynamics of loops may confer advantages over highly fixed folded proteins in substrate binding.^40^ The free energy flow can trade off attractive forces for the substrate bound form with the entropy of a loop, allowing for specificity but retaining a reasonable equilibrium constant for product release. The diffusive motions of a loop in thermal equilibrium with the environment might also help “pull” off the substrate from its post-transition state like environment. Many of these functionally important dynamics are retained in the crystalline forms of the protein as well. Their motions are just averaged by the nature of the analysis and are not well represented by a single model built based on standard practices. Compared to other methods of characterizing the energy landscape of a large dynamic molecule, ensemble refinement is an easier way of analyzing currently available structure data in protein databank and representing protein dynamics in atomic detail with enhanced support from experimental data.

The general fold of CalS11 is stable in structures with or without SAH bound. Loop L2 of CalS11 shows enhanced mobility in the absence of substrate. This mobility is visualized by ensemble refinement, which generated an ensemble of models by a restrained molecular dynamics simulation that includes the X-ray diffraction data. Ensemble refinement results of three structures showed that L2 conformations are distributed between closed and open states. Ensemble refinements provide us with a better representation of the mobile part of a protein structure, both statistically and visually. It helps us identify the spatial distribution of L2 for CalS11, presents a sampling of the conformational landscape, and provides evidence of dynamics of L2 supporting its function in promoting substrate binding and product release.
